# Kinematic and dynamic data from a robotic assembly of aeronautical threaded fasteners

**DOI:** 10.1016/j.dib.2023.109674

**Published:** 2023-10-14

**Authors:** Gustavo Jose Giardini Lahr, Thiago Henrique Segreto Silva, Guilherme Ribeiro Moreira, Thiago Boaventura, Glauco Augusto de Paula Caurin, Arash Ajoudani

**Affiliations:** a HRI2 Lab, Italian Institute of Technology, via San Quirico 19D, 16163 Genoa, Italy; b University of Sao Paulo, Av. Trabalhador Sao-Carlense 400, 13566-590 Sao Carlos, Brazil

**Keywords:** Robotics, Automation, Time series, Industrial assembly

## Abstract

Industrial screwing is one of several industry branches' most common manufacturing processes. Good quality and structured data from these operations have increased demand with the popularization of data-driven techniques for manufacturing automation. The dataset presented in this paper comprises screwing experiments with aeronautical nuts performed by an industrial robot Kuka KR-16 in a lab setting. The data comprises force, torque, linear and angular displacements, and velocities in time-series format. The dataset contains three different experiment results: mounted, jammed, and not mounted, which can be used as labels for classification techniques.

Specifications Table

**Table:** 

Subject	Engineering / Mechanical Engineering
Specific subject area	Dynamic and kinematic data for aeronautical manufacturing using industrial robot manipulators and compliant controller.
Data format	Raw
Type of data	Table
Data collection	A Kuka KR16 industrial robot inserts the nuts into the bolts, aligning and screwing them. It uses an interaction controller to avoid high forces/torques, runs a backspin (rotating in the opposite direction of the thread), and spins to finish the assembly. Data is acquired through a force-torque sensor affixed to the robotic manipulator's wrist and the log of the robot's kinematic parameters, i.e., position and orientation coordinates.
Data source location	University of Sao Paulo, Engineering School of Sao Carlos, Sao Carlos, Sao Paulo. Brazil
Data accessibility	Repository name: Mendeley Data [1] Data identification number: 10.17632/26674p3hvg.1 Direct URL to data: https://data.mendeley.com/datasets/26674p3hvg/1

## 1 Value of the Data

• The assembly of threaded fasteners is among the most used fastening processes in the industry, and the use of robots for this task in aircraft manufacturing has been increasing.
• This repository facilitates the development of techniques based on machine learning for state detection during the robotic assembly of threaded fasteners. It may benefit researchers and engineers focused on manufacturing large parts that demand a hole-in-peg configuration, such as aircraft, ships, and oil and gas tanks. In these cases, detecting the current assembly state is paramount to avoid rework or part removal.
• This data may be added to other datasets or used to pre-train neural networks for robotics assembly.

## 2 Data Description

As robots started to be deployed into semi-structured and unstructured environments, such as aircraft manufacturing and outdoors, developing techniques to improve their autonomy in these complex setups became necessary. Although the robot may have access to several sensor information to run in these scenarios, such as the actuators’ positions, velocities, forces, and torques, it is still challenging to state whether a specific task succeeded or failed. Thus, distinguishing between states during operation is paramount to properly designing the recovery procedures detrimental to such autonomy and performance enhancements [2].

**Fig. 1:**
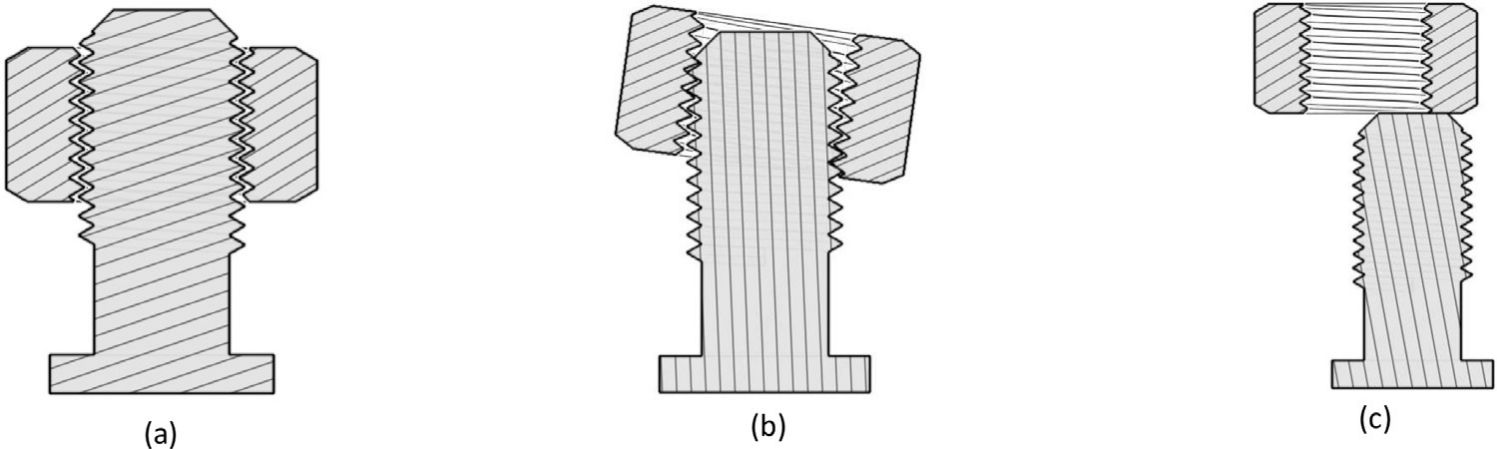
Three possible assembly outcomes of the nut during the assembly attempt:(a) mounted, (b) jammed, (c) not mounted.

Kinematic (e.g., position and velocity) and dynamic data (force and torque) can be combined with detection algorithms to differentiate dynamical states and detect failure. These data are commonly generated from industrial robots in most manufacturing scenarios, making them suitable for general detection systems. In this context, the dataset presented in this paper was generated from robotic screwing, a prevalent task in the manufacturing industry. It contains time sequence data for position, orientation, force, and torque related to threaded fasteners’ assembly with robotic manipulators and their respective outcomes. The outcomes were classified into the following groups (shown in Fig. 1):

1. **Mounted** - a successful assembly case where all geometric constraints were satisfied (1a);
2. **Jammed** - a situation where the nut would have an incorrect assembly, and it would demand high torques to remove it (1b);
3. **Not mounted** - when the alignment error is large enough to prevent a correct nut insertion into the bolt, or when the insertion is done correctly, and by the end of the screwing, the nut was not mounted correctly without developing high torques (1c).

**Fig. 2:**
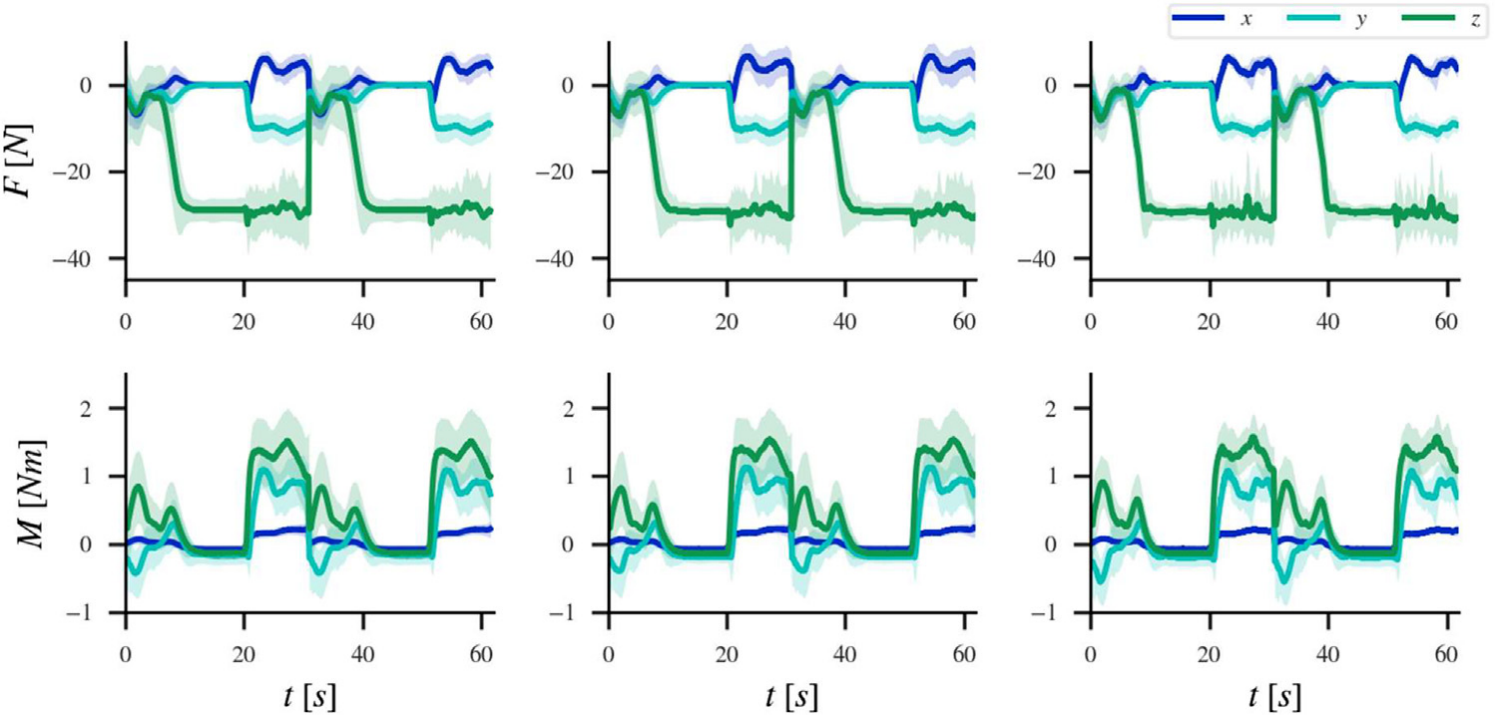
Forces (first row) and torques (second row) averages and their respective standard deviations for the different labels: Mounted (first column), Not Mounted (second column), and Jammed (third column).

The experiments presented in the dataset correspond to special aircraft nuts, also known as collars [3]. A total of 479 samples were collected to create a database, with 306 (63.9 %) of mounted cases, 112 (23.4 %) not mounted, and 61 (12.7 %) jammed. Fig. 2 shows the average force-torque profile for each class with the collar data after a synchronization at the first contact. The shaded area corresponds to the standard deviation for that time step.

### 2.1 Dataset organization

The folder “data” contains the time series for each experiment. The columns respect the order the Table 1 presents with their respective units. The file name follows a pattern: the first four numbers represent that experiment's batch, and the last three indicate which bolt that file was mounted on, as depicted below. Collars are accounted as a single batch, thus receiving 9999 as the batch number:

**Table 1:** Description of the .csv files content.

Variable	Units	Description
time	s	Time elapsed for that run
x, y, z	mm	Position of the end-effector in the world frame.
rotx, roty, rotz		Euler angles of the end-effector in the world frame^1^.
fx, fy, fz	N	Forces measured by the force-torque sensor in the sensor frame.
mx, my, mz	Nm	Torques measured by the force-torque sensor in the sensor frame.
vx, vy, vz	mm/s	Linear velocities of the end-effector in the world frame^2^.
vrotx, vroty, vrotz	^o^/s	Angular velocities of the end-effector in the world frame^2^.

1 Kuka robots use A, B, and C for consecutive rotations along with Z (rotz), Y (roty), and X (rotx) axes, respectively.

2 Calculated by finite difference of one single step.

The dataset has a ”meta.csv” file, which contains the description for each experiment of each run. The recorded parameters are described in Table 2.

**Table 2:** Description of the variables in the meta.csv file.

Variable	Type	Description
idx	Integer	The index of the bolt in the matrix of bolts.
individual_time	Numeric	Each experiment time.
label	Categorical	The outcome for that bolt.
experiment_id	Text	The corresponding csv file name.

## 3 Experimental Design, Materials and Methods

**Fig. 3:**
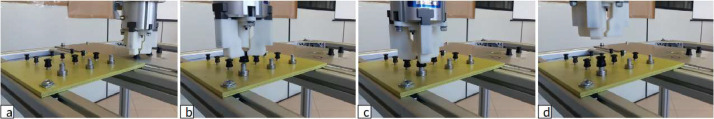
Steps of the assembly procedure executed by the robot: (a) pick the collar and position it on top of the bolt; (b) align the collar into the bolt using interaction controller; (c) backspin and screw; (d) release and evaluation of the assembly outcome.

A Kuka KR16 industrial robot assemblies the collars into the bolts in a procedure encompassing three phases: translational alignment, rotational fitting, and screwing, all depicted in Fig. 3. The translational alignment starts with the robotic system grasping a nut from a predetermined position (2a), moving it in proximity to a bolt (2b), and aligning the parts for mating using translational control. Notably, misalignments are intentionally introduced to induce more occurrences of not mounted and jammed cases: translational errors along the X and Y, sampled according to a Gaussian distribution $\varepsilon_{xy}∼N(\mu =0,\sigma =0.7\;mm$, and rotational misalignment along the x-axis (*Ĉ*in Kuka's notation), also Gaussian distributed with $\varepsilon_{\hat{c}}∼N(\mu =0,\sigma =0.3^{\circ }$. Upon reaching a position directly above the bolt, the planner activates the interaction controller, commencing the translational alignment phase. During this phase, the interaction controller manipulates the robot's end-effector until the desired force of -30 N in the z-direction is achieved while maintaining null forces in the xy-axes. From the instant the collar reaches the position to start the insertion up to the moment the robot reaches the desired force, it takes approximately 20 seconds.

Then, the second phase of rotational fitting starts (2c). The movement is named backspinning, where the nut is rotated opposite the threads to prepare it for accommodating the screw, and the robotic system backspins the nut by 300^o^. Subsequently, the third and final step entails the screwing process, rotating by 600^o^ to securely fasten the collar onto the bolt. Finally, the gripper opens, and the robot recovers for the next trial (2d).

**Fig. 4:**
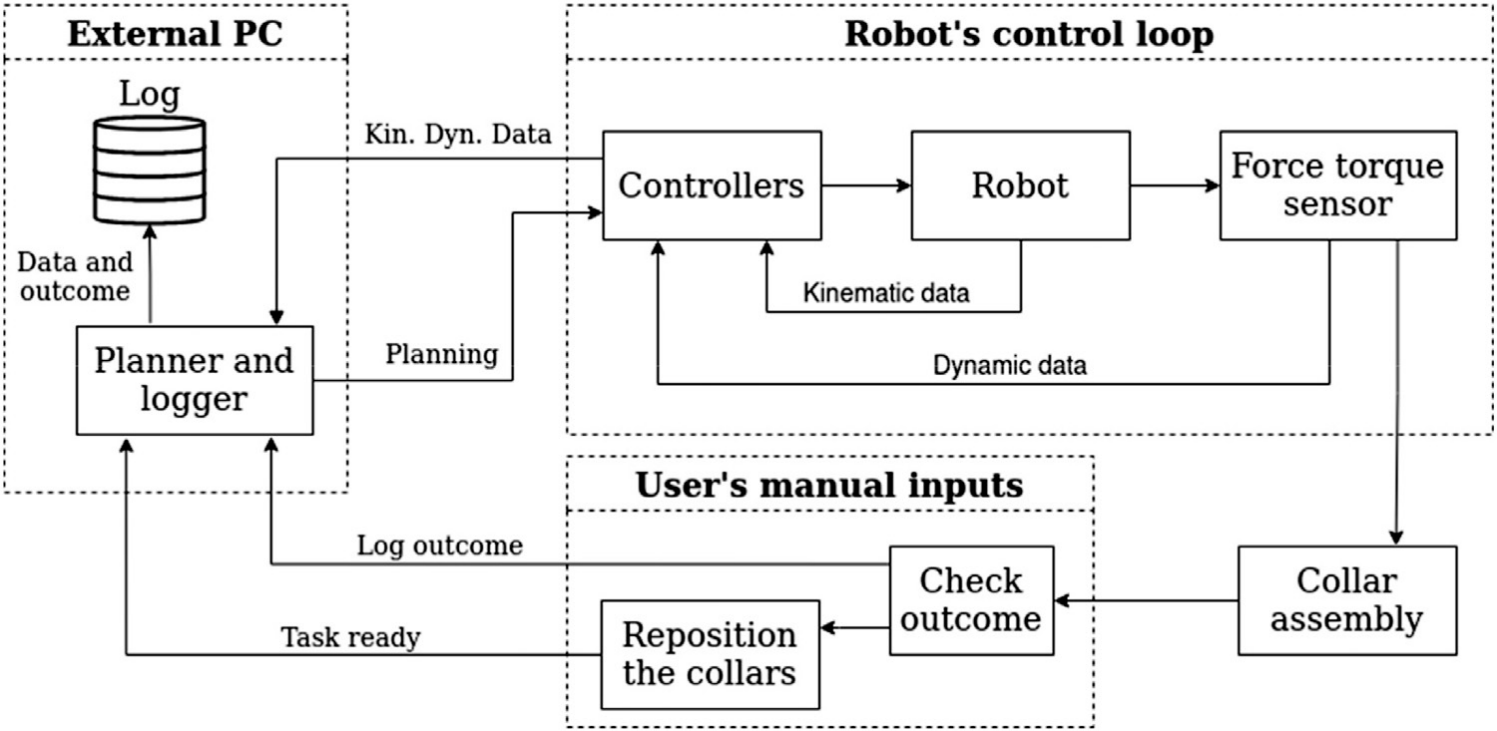
Communication between the planner within the external PC, robot's controller, and the user inputs.

Each trial takes up to 31 seconds to run, where position and force-torque data are recorded, generating each sample with 2560 time steps with 18 channels plus time. The sampler records the data within a 12 ms period. The tool is an SMC 3-finger pneumatic gripper, model MHS3-50D, and it is connected to a force-torque sensor, ATI-IA model Delta SI-660-60, mounted to the robot's wrist. After the assembly ends, the user runs a manual verification by checking the final state of the nut and recording it on a digital report for that bolt. Only the bolts are kept in the record of its index, and the nuts are randomly assembled. The data flow between the external planner, the robot's controller, and the user inputs with the task of collar assembly is shown in Fig. 4.

## Limitations

The collection of this dataset did not include forcing errors to obtain more data from minority classes, e.g., running assemblies without collars or not matching collar-bolt sizes. Therefore, it is an imbalanced dataset. Moreover, no data was collected in other configurations, e.g., vertical setups or in a workbench with different stiffness to simulate other aircraft parts.

## Ethics Statement

The authors have read and followed the ethical requirements for publication in Data in Brief. None of the data here published involves human subjects, animal experiments, or any data collected from social media platforms.

## CRediT authorship contribution statement

**Gustavo Jose Giardini Lahr:** Conceptualization, Writing – original draft, Data curation. **Thiago Henrique Segreto Silva:** Visualization, Writing – review & editing. **Guilherme Ribeiro Moreira:** Data curation, Investigation, Software. **Thiago Boaventura:** Supervision. **Glauco Augusto de Paula Caurin:** Writing – review & editing, Supervision, Funding acquisition. **Arash Ajoudani:** Writing – review & editing, Funding acquisition.

## Data Availability

Robotic assembly data of threaded fasteners: aeronautical collars (Original data) (Mendeley Data)

## Appendix Supplementary materials

Image, video 1
